# Placental Pathology Findings and the Risk of Intraventricular and Cerebellar Hemorrhage in Preterm Neonates

**DOI:** 10.3389/fneur.2020.00761

**Published:** 2020-08-14

**Authors:** Alessandro Parodi, Laura Costanza De Angelis, Martina Re, Sarah Raffa, Mariya Malova, Andrea Rossi, Mariasavina Severino, Domenico Tortora, Giovanni Morana, Maria Grazia Calevo, Maria Pia Brisigotti, Francesca Buffelli, Ezio Fulcheri, Luca Antonio Ramenghi

**Affiliations:** ^1^Neonatal Intensive Care Unit, Department Mother and Child, IRCCS Istituto Giannina Gaslini, Genoa, Italy; ^2^Department of Neurosciences, Rehabilitation, Ophthalmology, Genetics, Maternal and Child Health (DINOGMI), University of Genoa, Genoa, Italy; ^3^Neuroradiology Unit, IRCCS Istituto Giannina Gaslini, Genoa, Italy; ^4^Department of Health Sciences (DISSAL), University of Genoa, Genoa, Italy; ^5^Epidemiology and Biostatistics Unit, IRCCS Istituto Giannina Gaslini, Genoa, Italy; ^6^Gynaecologic and Fetal-Perinatal Pathology Centre, IRCCS Istituto Giannina Gaslini, Genoa, Italy; ^7^Division of Pathology, Department of Surgical Sciences (DISC), University of Genoa, Genoa, Italy

**Keywords:** placenta, intraventricular hemorrhage, cerebellar hemorrhage, preterm infant, chorioamnionitis, maternal malperfusion, magnetic resonance imaging

## Abstract

Placental pathology as a predisposing factor to intraventricular hemorrhage remains a matter of debate, and its contribution to cerebellar hemorrhage development is still largely unexplored. Our study aimed to assess placental and perinatal risk factors for intraventricular and cerebellar hemorrhages in preterm infants. This retrospective cohort study included very low-birth weight infants born at the Gaslini Children's Hospital between January 2012 and October 2016 who underwent brain magnetic resonance with susceptibility-weighted imaging at term-equivalent age and whose placenta was analyzed according to the Amsterdam Placental Workshop Group Consensus Statement. Of the 286 neonates included, 68 (23.8%) had intraventricular hemorrhage (all grades) and 48 (16.8%) had a cerebellar hemorrhage (all grades). After correction for gestational age, chorioamnionitis involving the maternal side of the placenta was found to be an independent risk factor for developing intraventricular hemorrhage, whereas there was no association between maternal and fetal inflammatory response and cerebellar hemorrhage. Among perinatal factors, we found that intraventricular hemorrhage was significantly associated with cerebellar hemorrhage (odds ratio [OR], 8.14), mechanical ventilation within the first 72 h (OR, 2.67), and patent ductus arteriosus requiring treatment (OR, 2.6), whereas cesarean section emerged as a protective factor (OR, 0.26). Inotropic support within 72 h after birth (OR, 5.24) and intraventricular hemorrhage (OR, 6.38) were independent risk factors for cerebellar hemorrhage, whereas higher gestational age was a protective factor (OR, 0.76). Assessing placental pathology may help in understanding mechanisms leading to intraventricular hemorrhage, although its possible role in predicting cerebellar bleeding needs further evaluation.

## Introduction

Despite significant improvements in the perinatal care of preterm infants, intraventricular hemorrhage (GMH-IVH), and cerebellar hemorrhage (CBH) still represent the most frequent lesions occurring during the very first few days of life (1–3). Besides ultrasonography, the implementation of magnetic resonance imaging (MRI) with susceptibility-weighted image (SWI) sequences as a diagnostic tool for preterm brain lesions has improved the accuracy in detecting minor forms of both supratentorial and infratentorial hemorrhages (4–7).

Both GMH-IVH and CBH have a multifactorial etiology (8–10), in which gestational age and perinatal factors seem to play a major role, although they have never been considered together in relation to placental risk factors. In the last decades, several studies have explored the contribution of the prenatal environment to the development of preterm brain lesions (11). In particular, the role of placental pathology in prematurity-related complications has been widely investigated but is still a matter of debate (12, 13). The heterogeneous results in different studies could be explained by the multiplicity of both placental lesion definition and classification.

In addition, whereas the majority of research has focused on GMH-IVH, to date, the contribution of placental pathology to CBH development is still largely unexplored. The aim of our study was to evaluate the role of placental histopathological features in the risk of developing GMH-IVH and CBH, which was diagnosed with brain MRIs performed at term-equivalent age (TEA) in a consecutive cohort of very low-birth weight (VLBW) infants. We hypothesized that the use of both a uniform and detailed placental histopathology classification and the use of brain MRI with SWI sequences to detect even minor forms of brain hemorrhage might better clarify the contribution of placental pathology for the development of both GMH-IVH and CBH in VLBW infants.

## Materials and Methods

The study was a retrospective analysis of a prospectively collected cohort of all preterm infants born with a birth weight of <1,500 g at Gaslini Children's Hospital (Genoa, Italy) between January 2012 and October 2016. Neonates were included if they underwent a brain MRI at TEA and had a placental pathology performed at birth. We excluded neonates with genetic syndromes, congenital malformations, and brain malformations. For GMH-IVH risk factor analysis, the case group included infants with MRI showing any grade of GMH-IVH, and for CBH risk factor analysis, the case group included infants with punctate (≤4 mm), limited (>4 mm but less than one-third of the cerebellar hemisphere), and massive (more than one-third of the cerebellar hemisphere) hemorrhage.

### Clinical Data Collection

Demographic and perinatal clinical data, including known risk factors for prematurity-related brain lesions, were collected by chart review. Prenatal variables included preeclampsia, number of fetuses, twin-to-twin transfusion, intrauterine growth restriction, antenatal steroids (two doses), gestational diabetes, and type of delivery (vaginal delivery or elective/emergent cesarean section). Neonatal variables included gestational age, gender, birth weight, 1′ and 5′ Apgar scores, surfactant administration, neonatal sepsis (early and late onset), necrotizing enterocolitis, and patent ductus arteriosus (PDA) requiring surgical or at least pharmacological treatment. Intubation, mechanical ventilation, pneumothorax, and inotropic treatments were recorded if they occurred within the first 72 h of life.

### Brain MRI

Brain MRI scans were obtained at TEA (between 39 and 41 weeks of corrected gestational age) according to our internal VLBW follow-up protocol. Scans were performed on a 1.5-Tesla system (Intera Achieva; Philips, Best, The Netherlands) using a pediatric dedicated head/spine coil. The exam was performed during natural sleep using the “feed and wrap” technique, whereas oral midazolam (0.1 mcg/kg) was used for mild sedation to prevent head motion in selected cases, according to the infant's state of arousal and image quality after the first sequence. Hearing protection was provided for all patients. Heart rate and oxygen saturation were monitored by pulse oximetry throughout the examination. MRI scans of the brain included 3-mm-thick axial T2- and T1-weighted images, 3-mm-thick coronal T2-weighted images, 3-mm-thick sagittal T1-weighted images, axial diffusion-weighted images (*b* value: 1,000 s/mm^2^), and axial SWIs. Informed consent that included statements about the significance and limitations of MRI at TEA was obtained in all cases.

In order to assess the presence of GMH-IVH and CBH, all images were reviewed by three neuroradiologists experienced in neonatal neuroimaging (DT, GM, and MS), who were unaware of perinatal history and placental pathology. Foci of signal loss on SWI alongside lateral ventricle walls or in the caudothalamic notch, without continuity suggestive of veins, were interpreted as intraventricular or subependymal hemosiderin depositions, consistent with previous GMH-IVH. Foci of signal loss on SWI within the cerebellum, without continuity suggestive of veins, were interpreted as hemosiderin depositions, consistent with previous CBH. Similar SWI findings within the fourth ventricle, potentially consistent with intraventricular blood of supratentorial origin, were not interpreted as CBH.

### Placental Pathology

Macroscopic examination results of each placenta were collected from the electronic database of our pathology department, and data about the presence of twin monochorionic placenta, velamentous placental cord insertion, and retroplacental hematoma were obtained from placental examination reports. Three experienced placental pathologists (EF, FB, and MB) reviewed placental sections for the microscopic analysis. The following formalin-fixed sections of placental parenchyma, of 3–5 μm thickness and stained with hematoxylin and eosin, were analyzed for every patient: two or more sections of extraplacental membrane roll, three cross sections of the umbilical cord, and 12 full-thickness sections of villous tissue, including one adjacent to the umbilical cord insertion site.

**Figure 1:**
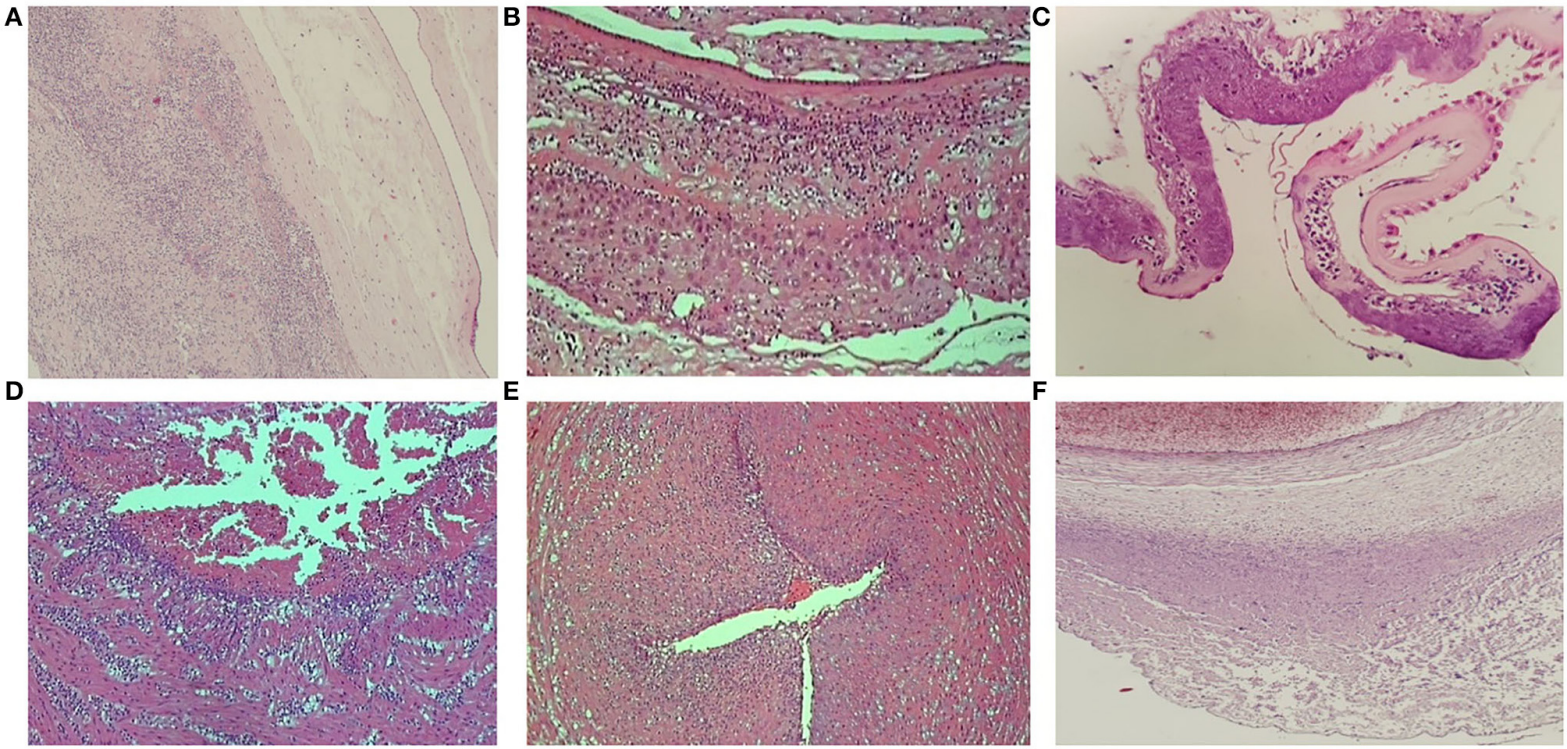
Placental sections showing the three stages of maternal and fetal inflammatory response. **(A)** Maternal inflammatory response—stage 1. **(B)** Maternal inflammatory response—stage 2. **(C)** Maternal inflammatory response—stage 3. **(D)** Fetal inflammatory response—stage 1. **(E)** Fetal inflammatory response—stage 2. **(F)** Fetal inflammatory response—stage 3.

Maternal and fetal stromal–vascular lesions were classified according to the Amsterdam Placental Workshop Group Consensus Statement (14), and the degree of maternal and fetal inflammatory response in ascending intrauterine infection was scored according to Redline et al. (15) and confirmed by the Amsterdam Placental Workshop Group Consensus Statement (14). Maternal inflammatory responses were classified as stage 1 (acute subchorionitis: patchy diffuse accumulations of neutrophils in the subchorionic plate and/or membranous chorionic trophoblast layer), stage 2 (acute chorioamnionitis: more than a few scattered neutrophils in the chorionic plate or membranous chorionic connective tissue and/or the amnion), or stage 3 (necrotizing chorioamnionitis: degenerating neutrophils, thickened eosinophilic amniotic basement membrane, and at least focal amnionic epithelial degeneration). Fetal inflammatory responses were classified as stage 1 (chorionic vasculitis/umbilical phlebitis: neutrophils in the wall of any chorionic plate vessel or the umbilical vein), stage 2 (umbilical vasculitis: neutrophils in one or both umbilical arteries and vein), or stage 3 (necrotizing funisitis or concentric umbilical perivasculitis: neutrophils, cellular debris, eosinophilic precipitate, and/or mineralization arranged in a concentric band, ring, or halo around one or more umbilical vessels) (Figure 1). Grade/intensity for both maternal and fetal inflammatory response was not included in the analysis.

Maternal vascular malperfusion was defined by the presence of the following microscopic features: accelerated villous maturation, distal villous hypoplasia, villous infarction, and decidual arteriopathy. Fetal vascular malperfusion was reported in cases of thrombosis, segmental avascular villi, intramural fibrin deposition, and villous stromal–vascular karyorrhexis. The presence of villitis of unknown etiology (multiple foci on more than one section, at least one of which showing inflammation affecting more than 10 contiguous villi) and delayed villous maturation (monotonous villous population with reduced numbers of vasculosyncytial membranes) were recorded.

## Statistical Analysis

Descriptive statistics were generated for the whole cohort, and data were expressed as mean and standard deviation for continuous variables. Median value and range were calculated and reported, as were absolute or relative frequencies for categorical variables. Demographic and clinical characteristics were compared using the χ^2^ or Fisher's exact test and the Student *t*-test for categorical and continuous variables, respectively. Univariate analysis was carried out to determine which demographic, perinatal, and placental characteristics were significantly more frequent among the patients with a specific lesion (GMH-IVH or CBH). Logistic regression analyses were used for each variable, and the results were reported as odds ratio (OR) with their 95% confidence intervals (CIs). The absence of exposure to the factor or the variable that was less likely to be associated with the risk of the lesion was used as the reference for each analysis. Multivariate analysis was then performed, and only variables that proved to be statistically or borderline significant in univariate analysis (*P* < 0.08) were included in the model. The model showing the best fit was based on backward stepwise selection procedures, and each variable was removed if it did not contribute significantly. In the final model, a *P* < 0.05 was considered statistically significant, and all *P*-values were based on two-tailed tests. Statistical analysis was performed using Statistical Package for the Social Sciences (SPSS) for Windows (SPSS Inc, Chicago, IL).

## Results

A total of 348 VLBW infants were identified across the study period. Of these, 26 (7.5%) patients died before reaching the TEA and one (0.3%) patient was excluded from the study because of the absence of brain MRI. Pathological examination of the placenta was not performed in 35 (10%) cases. The remaining 286 (82.2%) subjects were included in the study. Of the 286 patients in the final study population, 135 were male (47.2%). Mean gestational age was 28.2 weeks (standard deviation, *SD* ± 2.2 weeks), and mean birth weight was 1,040 g (*SD* ± 264 g). Median 1′ and 5′ Apgar scores were 6 (range, 0–9) and 8 (range, 0–10). GMH-IVH of any grade was detected in 68 patients (23.8%), whereas CHB was identified in 48 patients (16.8%). Of note, 50/68 infants (73.5%) with MRI-diagnosed GMH-IVH had already been diagnosed with cranial ultrasound, and the majority of them (47/50, 94%) received the diagnosis within the first 72 h of life. In addition, in the group of patients excluded because of the absence of placental examination, we found no significant difference in the incidence of GMH-IVH (10/35, 28.6%) nor CBH (4/35, 11.4%) compared to the study population (*p* = 0.677 and 0.626, respectively).

### Risk Factors for GMH-IVH

Demographic and perinatal clinical data are shown in Table 1. Mean gestational age (27 ± 2.2 vs. 28.6 ± 2.1 weeks; *P* = 0.001) and birth weight (908 ± 250 vs. 1,080 ± 255 g; *P* = 0.001) were significantly lower in patients who developed GMH-IVH. In the univariate analysis, infants with GMH-IVH were less likely to be exposed to preeclampsia and delivered by cesarean section (*P* = 0.04 and *P* ≤ 0.0001, respectively). Infants with GMH-IVH had a significantly higher frequency of 5′ Apgar score lower than five, intubation, mechanical ventilation, and inotropic support within the first 72 h, surfactant, late-onset sepsis, and medically and surgically treated PDA (Table 1). In addition, the contemporary presence of CBH was significantly more frequent in this population (*P* = 0.0001).

**Table 1:** Demographic information, clinical characteristics, and placental findings of neonates with and without intraventricular hemorrhage (GMH-IVH).

	**Study population (*N* = 286)**	**GMH-IVH (*N* = 68)**	**No GMH-IVH (*N* = 218)**	***P*-value**
	***N* (%)**	***N* (%)**	***N* (%)**	
**Prenatal data**				
Male	135 (47.2)	36 (52.9)	99 (45.4)	0.33
Intrauterine growth restriction	78 (27.3)	15 (22.1)	63 (28.9)	0.35
Antenatal corticosteroids	224 (78.6)	48 (70.6)	176 (80.7)	0.09
Gestational diabetes	11 (3.8)	2 (2.9)	9 (4.1)	1
Preeclampsia	64 (22.4)	9 (13.2)	55 (27.1)	**0.04**
Twin pregnancy	113 (39.5)	28 (41.2)	85 (39.0)	0.78
Twin to twin transfusion	22 (7.7)	5 (7.4)	17 (7.8)	1
Cesarean section	227 (79.4)	43 (63.2)	184 (84.4)	**≤0.0001**
**Postnatal data**				
Gestational age at birth mean (*SD*), weeks	28.2 ± 2.2	27 ± 2.2	28.6 ± 2.1	0.001
Birth weight mean (*SD*), g	1040 ± 264	908 ± 250	1080 ± 255	0.001
1′ Apgar ≤ 5	114 (39.9)	34 (50)	80 (36.7)	0.06
5′ Apgar ≤ 5	11 (3.8)	7 (10.3)	4 (1.8)	**0.005**
Intubation within the first 72 h	205 (71.7)	61 (89.7)	144 (66.1)	**<0.0001**
Mechanical ventilation within the first 72 h	149 (52.1)	49 (72.1)	100 (45.9)	**<0.0001**
Surfactant administration	200 (69.9)	59 (86.8)	141 (64.7)	**<0.0001**
Pneumothorax within the first 72 h	11 (3.8)	4 (5.9)	7 (3.2)	0.29
Inotropic support within the first 72 h	24 (8.4)	10 (14.7)	14 (6.4)	**0.04**
Early-onset sepsis	10 (3.5)	3 (4.4)	7 (3.2)	0.71
Late-onset sepsis	131 (45.8)	39 (57.4)	92 (42.2)	**0.03**
>1 late-onset sepsis	44 (15.4)	17 (25)	27 (12.4)	**0.02**
Necrotizing enterocolitis	17 (5.9)	5 (7.4)	12 (5.5)	0.56
Surgically treated necrotizing enterocolitis	10 (3.5)	4 (5.9)	6 (2.8)	0.25
Treated patent ductus arteriosus	174 (60.8)	51 (75)	123 (56.4)	**0.007**
Surgically treated patent ductus arteriosus	31 (10.8)	16 (23.5)	15 (6.9)	**<0.0001**
Cerebellar hemorrhage	48 (16.8)	30 (44.1)	18 (8.3)	**0.0001**
**Placental findings**				
Twin monochorionic placenta	38 (13.3)	7 (10.3)	31(14.2)	0.54
Velamentous placental cord insertion	27 (9.4)	5 (7.4)	22 (10.1)	0.64
Fetal inflammatory response—stage 1	23 (8)	9 (13.2)	14 (6.4)	0.09
Fetal inflammatory response—stage 2	9 (3.1)	5 (7.4)	4 (1.8)	**0.04**
Fetal inflammatory response—stage 3	23 (8)	6 (8.8)	17 (7.8)	0.80
Maternal inflammatory response—stage 1	29 (10.1)	11 (16.2)	18 (8.3)	0.06
Maternal inflammatory response—stage 2	37 (12.9)	8 (11.8)	29 (13.3)	0.84
Maternal inflammatory response—stage 3	22 (7.7)	11 (16.2)	11 (5)	**0.007**
Villitis of unknown etiology	31 (10.8)	10 (14.7)	21 (9.6)	0.26
Maternal vascular malperfusion	165 (57.7)	32 (47.1)	133 (61)	**0.05**
Fetal vascular malperfusion	11 (3.8)	3 (4.4)	8 (3.7)	0.73
Delayed villous maturation	44 (15.4)	8 (11.8)	36 (16.5)	0.26
Retroplacental hematoma	52 (18.2)	12 (17.6)	40 (18.3)	1

*Statistically significant differences (p < 0.05) are highlighted in bold*.

Placental findings of the two populations are shown in Table 1. Across the spectrum of histological chorioamnionitis, in the univariate analysis, severe maternal inflammatory response (stage 3) and fetal inflammatory response (stage 2) were associated with GMH-IVH (*P* = 0.007 and *P* = 0.04, respectively). Conversely, maternal vascular malperfusion was more frequent in infants without this lesion (*P* = 0.05).

As shown in Table 2, risk factors for GMH-IVH identified by multivariate analysis adjusted for gestational age were the presence of CBH (OR, 8.14; 95% CI, 3.63–18.24), mechanical ventilation within the first 72 h (OR, 2.67; 95% CI, 1.23–5.79), and pharmacologically treated PDA (OR, 2.6; 95% CI, 1.16–5.83). In our cohort, both mild (stage 1) and severe (stage 3) maternal inflammatory responses were found to be an independent risk factor for developing GMH-IVH (OR, 2.92; 95% CI, 1.04–8.19 and OR, 4; 95% CI, 1.24–12.9, respectively). Cesarean section was a protective factor (OR, 0.26; 95% CI, 0.11–0.58).

**Table 2:** Logistic regression analysis of potential risk factors for intraventricular hemorrhage (GMH-IVH).

	**GMH-IVH (*N* = 68)**	**No GMH-IVH (*N* = 218)**	**OR (95% CI)**	***P*-value**
	***N* (%)**	***N* (%)**		
Cesarean section	43 (63.2)	184 (84.4)	0.26 (0.11–0.58)	**<0.001**
Maternal inflammatory response—stage 1	11 (16.2)	18 (8.3)	2.92 (1.04–8.19)	**0.04**
Maternal inflammatory response—stage 3	11 (16.2)	11 (5)	4 (1.24–12.9)	**0.02**
Treated patent ductus arteriosus	51 (75)	123 (56.4)	2.6 (1.16–5.83)	**0.02**
Mechanical ventilation within the first 72 h	49 (72.1)	100 (45.9)	2.67 (1.23–5.79)	**0.01**
Cerebellar hemorrhage	30 (44.1)	18 (8.3)	8.14 (3.63–18.24)	**<0.001**

*OR, odds ratio; CI, confidence interval. Statistically significant differences (p < 0.05) are highlighted in bold*.

### Risk Factors for Cerebellar Hemorrhage

Mean gestational age (26.6 ± 2 vs. 28.6 ± 2.1 weeks) and birth weight (904 ± 254 vs. 1,067 ± 258 g) were significantly lower in neonates with CBH (*P* = 0.0001). By univariate analysis, intubation, mechanical ventilation, surfactant, hemodynamically significant PDA, inotropic support, late-onset sepsis, and GMH-IVH were significantly more frequent (Table 3). Among placental factors, neither maternal or fetal vascular malperfusion nor histologic chorioamnionitis (both maternal and fetal sides) was associated with this lesion in our cohort. In the multivariate analysis (Table 4), lower gestational age, inotropic support within 72 h after birth (OR, 5.24; 95% CI, 1.88–14.6), and the contemporary presence of GMH-IVH (OR, 6.38; 95% CI, 3.02–13.5) emerged as independent risk factors for CBH.

**Table 3:** Demographic information, clinical characteristics, and placental findings of neonates with and without cerebellar hemorrhage (CBH).

	**Study patients (*N* = 286)**	**CBH (*N* = 48)**	**No CBH (*N* = 238)**	***P*-value**
	***N* (%)**	***N* (%)**	***N* (%)**	
**Prenatal data**				
Male	135 (47.2)	26 (54.2)	109 (45.8)	0.34
Intrauterine growth restriction	78 (27.3)	8 (16.7)	70 (29.4)	0.08
Antenatal steroids	224 (78.6)	32 (66.7)	192 (80.7)	**0.04**
Gestational diabetes	11 (3.8)	1 (2.1)	10 (4.2)	0.70
Preeclampsia	55 (19.2)	8 (16.7)	56 (23.5)	0.35
Twin pregnancy	110 (38.5)	19 (39.6)	91 (38.2)	0.87
Twin-to-twin transfusion	227 (79.4)	35 (72.9)	192 (80.7)	0.24
Cesarean section				
**Postnatal data**				
Gestational age at birth mean (*SD*), weeks	28.2 ± 2.2	26.6 ± 2	28.6 ± 2.1	**0.0001**
Birth weight mean (*SD*), g	1040 ± 264	904 ± 254	1067 ± 258	**0.0001**
1′ Apgar ≤ 5	114 (39.9)	23 (47.9)	91 (38.2)	0.26
5′ Apgar ≤ 5	11 (3.8)	3 (6.2)	8 (3.4)	0.40
Intubation within the first 72 h	205 (71.7)	43 (89.6)	162 (68.1)	**0.002**
Mechanical ventilation within the first 72 h	149 (52.1)	32 (66.7)	117 (49.2)	**0.03**
Surfactant administration	200 (69.9)	43 (89.6)	157 (66)	**0.001**
Pneumothorax within the first 72 h	11 (3.8)	3 (6.2)	8 (3.4)	0.40
Inotropic support within the first 72 h	24 (8.4)	13 (27.1)	11 (4.6)	**0.0001**
Early-onset sepsis	10 (3.5)	3 (6.2)	7 (2.9)	0.38
Late-onset sepsis	131 (45.8)	34 (70.8)	97 (40.8)	**0.0001**
>1 late-onset sepsis	44 (15.4)	14 (29.2)	30 (12.6)	**0.007**
Necrotizing enterocolitis	17 (5.9)	3 (6.2)	14 (5.9)	1
Surgically treated necrotizing enterocolitis	10 (3.5)	3 (6.2)	7 (2.9)	0.38
Treated patent ductus arteriosus	174 (60.8)	32 (66.7)	142 (59.7)	0.42
Surgically treated patent ductus arteriosus	31 (10.8)	13 (27.1)	18 (7.6)	**0.0001**
GMH-IVH	68 (23.8)	30 (62.5)	38 (18)	**0.0001**
**Placental findings**				
Twin monochorionic placenta	38 (13.3)	4 (8.3)	34(14.3)	0.35
Velamentous cord insertion	27 (9.4)	1 (2.1)	26 (10.9)	0.06
Fetal inflammatory response—stage 1	23 (8)	4 (8.3)	19 (8)	1
Fetal inflammatory response—stage 2	9 (3.1)	2 (4.2)	7 (2.9)	0.65
Fetal inflammatory response—stage 3	23 (8)	7 (14.6)	16 (6.7)	0.08
Maternal inflammatory response—stage 1	29 (10.1)	8 (16.7)	21 (8.8)	0.12
Maternal inflammatory response—stage 2	37 (12.9)	8 (16.7)	29 (12.2)	0.48
Maternal inflammatory response—stage 3	22 (7.7)	6 (12.5)	16 (6.7)	0.23
Villitis of unknown etiology	31 (10.8)	4 (8.3)	27 (11.3)	0.80
Fetal vascular malperfusion	11 (3.8)	1 (2.1)	10 (4.2)	0.70
Maternal vascular malperfusion	11 (3.8)	1 (2.1)	10 (4.2)	0.70
Delayed villous maturation	44 (15.4)	7 (14.6)	37 (15.5)	1
Retroplacental hematoma	52 (18.2)	11 (22.9)	41 (17.2)	0.41

*Statistically significant differences (p < 0.05) are highlighted in bold*.

**Table 4:** Logistic regression analysis of potential risk factors for cerebellar hemorrhage (CBH) in our cohort.

	**CBH (*N* = 48)**	**No CBH (*N* = 238)**	**OR (95% CI)**	***P*-value**
	***N* (%)**	***N* (%)**		
Gestational age	26.6 ± 2	28.6 ± 2.1	0.76 (0.63–0.91)	**0.003**
Inotropic support within the first 72 h	13 (27.1)	11 (4.6)	5.24 (1.88–14.6)	**0.002**
GMH-IVH	30 (62.5)	38 (18)	6.38 (3.02–13.5)	**0.0001**

*OR, odds ratio; CI, confidence interval. Statistically significant differences (p < 0.05) are highlighted in bold*.

## Discussion

Over the last decades, advances in perinatal care have contributed to a consistent reduction in severe forms of prematurity-related brain injuries such as periventricular leukomalacia (16). Nevertheless, the same efforts have not been as effective in reducing the incidence of GMH-IVH, which remains a common brain lesion in preterm infants, affecting about one-third of neonates of <32 weeks' gestation (17, 18). Extremely preterm infants are also prone to CBH, and after implementation of MRI for studying neonatal brain, the reported prevalence of CBH in low-birth weight infants went from 2% to a maximum of nearly 20% (6, 19, 20). Both lesions have a significant negative impact on neurodevelopmental outcome in preterm infants (21), although the diagnosis of minor forms of hemorrhage is often challenging with ultrasound and conventional MRI (4, 22) and how these lesions affect neurodevelopment is still a matter of debate (23, 24).

The multifactorial etiology and the relatively small development time frame of GMH-IVH and CBH make their prevention extremely challenging (25). For this reason, many efforts have been made to deeply understand their etiopathology as well as their associated risk factors. A complex interaction between environmental and genetic factors contributes to intraventricular hemorrhage in preterm infants (26). The role of genetic factors has recently gained interest, although the association with the development of GMH-IVH still remains unclear (27). Studies investigating the role of thrombophilic disorders increasing the risk of developing GMH-IVH are consistent with the phenomenon of thrombosis in germinal matrix vessels as a “primum movens” (10, 28–31), although conflicting results have been also published (32). At the same time, many studies found an increased risk of GMH-IVH in neonates with gene polymorphisms of pro-inflammatory cytokines, including IL-1β, IL-6, and TNF-α and of genes associated with the regulation of systemic blood pressure and cerebral blood flow, such as endothelial nitric oxide synthase (33) and type IV collagen genes (34).

Gestational age and birth weight play a major role in the development of GMH-IVH and CBH, and the lower the weight and gestational age, the higher their incidence (19, 35). Our study showed a strong relationship between low gestational age at birth and CBH, in which the risk of this lesion was reduced at higher gestational ages.

The fetal environment is likely to influence the development of GMH-IVH and CBH because of the precocity of their occurrence. As placental macroscopic and histologic features reflect the quality of intrauterine life, several studies have focused on placental pathology in order to assess whether an association exists between placental lesions and the risk of developing neonatal brain injuries (12). Intrauterine inflammation/infection (chorioamnionitis) is among the most studied placental lesions associated with preterm birth and potentially with preterm-related complications (36).

The relationship between chorioamnionitis and the development of GMH-IVH is still a matter of debate. A study in 2012 failed to find any association (37), whereas the meta-analysis of Villamor-Martinez et al. (13) showed that both clinical and histological chorioamnionitis constitute an independent risk factor for GMH-IVH. In 2018, Granger et al. demonstrated the same association, although after adjustment for perinatal variables, this association disappeared (38). In the same year, a multicenter study on 350 preterm infants found no association between histological chorioamnionitis and GMH-IVH diagnosed with early postnatal brain MRI (39). Heterogeneous methodology of the studies, different criteria for chorioamnionitis and brain damage diagnosis and staging, and continuous improvement in the clinical care of preterm infants (e.g., the introduction of antenatal steroid prophylaxis) could be possible explanations for this discrepancy (40).

We observed that both mild and severe (stages 1 and 3) histopathologic chorioamnionitis involving the maternal side of the placenta were independent risk factors for the development of GMH-IVH. We observed that both stage 1 and stage 3 histopathologic chorioamnionitis were independent risk factors for the development of GMH-IVH, while stage 2 chorioamnionitis did not show any significant association with GMH-IVH in our cohort.

Besides, fetal inflammatory response, characterized by chorionic vasculitis and different stages of funisitis, was significantly more frequent in the GMH-IVH group only in moderate forms (stage 2 fetal inflammatory response), but this association disappeared in the multivariate analysis. The causal relationship between chorioamnionitis and GMH-IVH is still unclear (41, 42), although plausible mechanisms include the direct effect of pro-inflammatory cytokines on the brain (43, 44), the increased permeability of the brain–blood barrier (45), and the augmented cerebral oxygen consumption associated with antenatal infection/inflammation (46–48).

In our study, the maternal component of intrauterine infection seemed to play a major role in enhancing the risk of GMH-IVH regardless of the presence of fetal inflammatory response. Our finding is consistent with the meta-analysis of Villamor Martinez et al., who evaluated the effect of funisitis on the development of intraventricular hemorrhage. In an analysis of 13 studies regarding infants with histological chorioamnionitis with or without funisitis, the authors did not find a significant difference in GMH-IVH risk between these two groups (13).

Maternal vascular malperfusion is a common placental finding in preterm birth (49) and may be associated with preeclampsia, stillbirth, intrauterine growth retardation, systemic lupus erythematosus, and antiphospholipid antibody syndrome (50). The placenta of nearly half of the VLBW infants in our cohort showed signs of maternal vascular malperfusion. In the univariate analysis, infants with GMH-IVH were less likely to present with this placental feature, but after correction for gestational age, this association disappeared.

In our cohort, histopathologic lesions of the placenta were not associated with a higher risk of CBH. The lack of association between cerebellar insult and chorioamnionitis is consistent with a recent meta-analysis, which included five studies in which clinically diagnosed chorioamnionitis was evaluated as a possible risk factor for CBH (51).

Although predisposition to GMH-IVH and CBH may potentially follow common perinatal patterns, it is possible that a different vascular anatomy may partly modify the factors, making these two brain regions predisposed to hemorrhage. Although subependymal vein anatomy may facilitate venous congestion leading to GMH-IVH (52), cerebellar vascular anatomy has not been investigated in detail as a potential factor contributing to CBH. In addition, human cerebellar development extends from the early first trimester to final circuit maturity, which is achieved by the end of the second postnatal year (53). The protracted nature of human cerebellar development renders this organ particularly vulnerable to developmental injury after birth, in which postnatal preterm-related complications may heavily impact its occurrence (4, 54), while antenatal placental disturbances may not significantly predispose infants to this lesion.

Among perinatal and postnatal risk factors, we found that cesarean section exerts a protective role on the development of both GMH-IVH and CBH compared to vaginal delivery. The same conclusion regarding GMH-IVH has emerged from two recent studies, in which elective cesarean section was found to reduce the rates of GMH-IVH in large cohorts of preterm infants born before 32 weeks' gestation (11, 55). At the same time, emergent cesarean section seems to increase the risk of CBH (19, 56). Elective cesarean section improving preterm neonatal outcome may be explained by the advantages related to a planned preterm birth, including an increased chance to administer a complete corticosteroid and antibiotic prophylaxes before birth, more attentive monitoring of fetal conditions, and perhaps, a more efficient preparation of the neonatal resuscitation team (57, 58). Nevertheless, as we included both emergent and elective sections, we are not able to confirm these associations.

Both circulatory and respiratory complications soon after birth predispose infants to GMH-IVH. In very preterm infants, cardiopulmonary resuscitation (35, 59–62), an increased number of intubation attempts in the delivery room (63), and mechanical ventilation (11, 64) were found to be risk factors for severe GMH-IVH. In addition, several studies have reported that neonatal hypotension increases the risk of both GMH-IVH and CBH in extremely preterm infants in the 1st day of life (65–67), possibly because of the immature cerebral autoregulation that may affect the preterm brain (68). Our study confirmed that a difficult adaptation to extrauterine life, demonstrated by a 5′ Apgar score <5, the presence of hemodynamic instability caused by patency of ductus arteriosus, hypotension requiring inotropic support, and the need for surfactant administration, and intubation and mechanical ventilation in the 1st days of life are likely to predispose the infant to both GMH-IVH and CBH. As reported in previous studies on very preterm and extremely preterm infants (11, 69), postnatal sepsis predisposed infants to both GMH-IVH and CBH in our cohort, although its effect was mitigated when corrected for gestational age. In the multivariate analysis, pharmacologically treated PDA and mechanical ventilation within the first 72 h remained significant factors predisposing infants to GMH-IVH, whereas hypotension requiring inotropic support within the first 72 h of life was an independent risk factor for CBH.

Although predisposing factors for GMH-IVH and CBH appear to be slightly different between our cohort and previous studies (56), the chance to have both lesions is high (OR of having a GMH-IVH in presence of CBH was 6.38 in our population and OR of having a CBH in case of GMH-IVH was 8.14 in our population). We suggest that preterm infants at risk for one lesion should be monitored for both GMH-IVH and CBH; interestingly, cerebral and cerebellar anatomical origins of the bleeding share a similar function (3), germinal matrix for GMH-IVH, and external granular layer for CBH, areas where neurons are produced prior to take their final migratory destiny. Of note, the major part of neuronal migration has already taken place in the germinal matrix when GMH-IVH may occur, whereas the external granular layer remains active in healthy infants until a few months after birth (53, 70).

A strength of our study was the use of MRI with SWI sequences to detect even minor forms of hemorrhage that may escape detection not only by ultrasonography but also by conventional magnetic resonance studies (71). In addition, we considered both forms of hemorrhage, GMH-IVH and CBH. As there is an urgent need for standardizing placental findings, we reviewed placental sections of every neonate included according to the recent histologic classification of the Amsterdam Placental Workshop Group Consensus Statement (14), which provides a detailed and comprehensive description of macroscopic and microscopic placental lesions.

Limitations of our study were its retrospective nature and the exclusion of all the patients who died before undergoing brain MRI at TEA or without a placental histologic examination performed at birth. This may have excluded the most preterm and sick neonates in which high-grade GMH-IVH and CBH are common complications. Due to the retrospective nature of the study, we could not identify the reasons why the placental examination was not performed. However, the prevalence of both GMH-IVH and CBH in this subgroup of subjects excluded from the analysis was similar to the prevalence we found in the study population.

Our study stresses the importance of postnatal care in the early neonatal period in reducing the risk of GMH-IVH and CBH. In addition, our data confirm that the presence of intrauterine infection/inflammation may play a significant role in predisposing preterm infants to GMH-IVH and reinforce the importance of preventing prenatal infections. Furthermore, to our knowledge, this is the first study exploring the possible association between placental pathology and hemorrhages like GMH-IVH and CBH, diagnosed, and considered together with SWI sequences. Despite the intuitive role of prenatal influence in early neonatal lesions such as GMH-IVH and CBH, the multifactorial etiology of these lesions and the strong influence of early postnatal factors may modulate individual effects of the prenatal environment on the subsequent risk of GMH-IVH and CBH. We believe that the best way to discover more significant risk factors differentiating the origin of the two lesions should rely on prospective and multicenter studies comparing selected cases of GMH-IVH (in the absence of CBH) to the even fewer isolated CBH.

## Data Availability Statement

All datasets generated for this study are included in the article/supplementary material.

## Ethics Statement

The studies involving human participants were reviewed and approved by Giannina Gaslini Hospital, Genoa, Italy. Written informed consent to participate in this study was provided by the participants' legal guardian/next of kin.

## Author Contributions

AP, LR, and EF contributed to conception and design of the study. AP, MR, SR, and LD organized the database. DT, AR, GM, and MS reviewed the MRI scans. EF, MB, and FB performed the placental analysis. MC performed the statistical analysis. AP and MR wrote the first draft of the manuscript. LD, FB, MC, MM, and LR wrote the sections of the manuscript. All authors contributed to manuscript revision, read, and approved the submitted version.

## Conflict of Interest

The authors declare that the research was conducted in the absence of any commercial or financial relationships that could be construed as a potential conflict of interest.
